# Trough Concentrations of Vancomycin in Patients Undergoing Extracorporeal Membrane Oxygenation

**DOI:** 10.1371/journal.pone.0141016

**Published:** 2015-11-06

**Authors:** So Jin Park, Jeong Hoon Yang, Hyo Jung Park, Yong Won In, Young Mi Lee, Yang Hyun Cho, Chi Ryang Chung, Chi-Min Park, Kyeongman Jeon, Gee Young Suh

**Affiliations:** 1 Department of Pharmaceutical Services, Samsung Medical Center, Seoul, Korea; 2 Department of Critical Care Medicine, Samsung Medical Center, Sungkyunkwan University School of Medicine, Seoul, Korea; 3 Division of Cardiology, Department of Medicine, Samsung Medical Center, Sungkyunkwan University School of Medicine, Seoul, Korea; 4 Department of Thoracic and Cardiovascular Surgery, Samsung Medical Center, Sungkyunkwan University School of Medicine, Seoul, Korea; 5 Division of Pulmonary and Critical Care Medicine, Department of Medicine, Samsung Medical Center, Sungkyunkwan University School of Medicine, Seoul, Korea; Medizinische Hochschule Hannover, GERMANY

## Abstract

To investigate the appropriateness of the current vancomycin dosing strategy in adult patients with extracorporeal membrane oxygenation (ECMO), between March 2013 and November 2013, patients who were treated with vancomycin while on ECMO were enrolled. Control group consisted of 60 patients on vancomycin without ECMO, stayed in medical intensive care unit during the same study period and with the same exclusion criteria. Early trough levels were obtained within the fourth dosing, and maintenance levels were measured at steady state. A total of 20 patients were included in the analysis in ECMO group. Sixteen patients received an initial intravenous dose of 1.0 g vancomycin followed by 1.0 g every 12 hours. The non-steady state trough level of vancomycin after starting administration was subtherapeutic in 19 patients (95.00%) in ECMO group as compared with 40 patients (66.67%) in the control group (*p* = 0.013). Vancomycin clearance was 1.27±0.51 mL/min/kg, vancomycin clearance/creatinine clearance ratio was 0.90 ± 0.37, and elimination rate constant was 0.12 ± 0.04 h^-1^. Vancomycin dosingfrequency and total daily dose were significantly increased after clinical pharmacokinetic services of the pharmacist based on calculated pharmacokinetic parameters (from 2.10 ± 0.72 to 2.90 ± 0.97times/day, *p* = 0.002 and from 32.54 ± 8.43 to 42.24 ± 14.62mg/kg, *p* = 0.014) in ECMO group in contrast with those (from 2.11 ± 0.69 to 2.37 ± 0.86 times/day, *p* = 0.071 and from 33.91 ± 11.85 to 31.61 ± 17.50 mg/kg, *p* = 0.350) in the control group.Although the elimination rate for vancomycin was similar with population parameter of non ECMO patients, the current dosing strategy of our institution for vancomycinin our ICU was not sufficient to achieve the target trough in the initial period in most patients receiving ECMO.

## Introduction

Although the technology and application of extracorporeal membrane oxygenation (ECMO) have progressed rapidly in recent years, appropriate drug dosing during ECMO has not been well established. In particular, sequestration of drugs in the circuit, increased volume of distribution, and decreased clearance are additional pharmacokinetic factors in the setting of extracorporeal circulation[1]. The immediate effect of acute hemodilution is a decrease in the total blood concentration of any drug present[2, 3]. In addition, the pharmacological impact will be larger if the drug has a small volume of distribution. Vancomycin is an essential antibiotic that is active against gram-positive bacteria, including methicillin-resistant staphylococci (MRSA), in the intensive care unit (ICU) setting. Its pharmacokinetic parameters can be presumed to interrelate with ECMO because the hydrophilic property of vancomycin affects its concentration in the body with the increased volume of distribution due to the application of ECMO. According to the recent guidelines, most of ICU patients undergoing ECMO need the therapeutic drug concentration of 15–20 mcg/ml[4, 5]. Therefore, in our institution, we check steady state trough levelnear the fifth dose not to be subtherapeutic and perform a clinical pharmacokinetic service if there is any need for dosage modification. However, for severely infectious patients such as ICU patients, the fifth dose is somewhat late for re-dosing because it sometimes takes more than two days for drug levels to stabilize. A subtherapeutic level during the initial period presents a great disadvantage to the treatment of sepsis, in which early effective antimicrobial administration is vital[6].Therefore, we investigated whether the current initial dosing of vancomycin based on total body weight and renal function is adequate in critically ill patients undergoing ECMO.

## Methods

### Study population

This study received Institutional Review Board approval (No. 2013-08-071-001 of Samsung Medical Center), and informed consent was waived for this retrospective study.Between March 2013 and November 2013, patients who were treated with vancomycin while on ECMO were enrolled in a retrospective, observational registry. We reviewed only the patients to whom ECMO and vancomycin were simultaneously applied. So all of the study subjects were on ECMO all the time during the vancomycin period in this study.Capiox Emergency Bypass System (Capiox EBS^TM^; Terumo Inc., Tokyo, Japan) and Permanent Life Support (PLS; MAQUET, Germany) were used in our hospital. These systems are composed of a portable controller with a back-up battery, a disposable bypass circuit integrated with a heparin-coated membrane oxygenator, and a centrifugal pump. Control group was defined as patients on vancomycin without ECMO, stayed in medical ICU during the same study period and with the same exclusion criteria. Exclusion criteria for the present analysis were: 1) 18 years of age or younger; 2) lack of clinical pharmacokinetic service records; 3) end-stage renal disease with ongoing any type of renal replacement therapy; 4) initial dosage not in the usual dosage range with respect to the patient’s renal function and body weight according to the Lexicomp® online dosage guidelinesbased on the 2009 consensus[4].

### Pharmacokinetic data collection

In our institution, we do not routinely administer vancomycin when ECMO is inserted. Only under clinical suspicion of infection, we start antibiotics. Pharmacokinetic service records were kept by the resident ICU pharmacist in charge of reviewing drug use in the respective ICU. When vancomycin is administered, a pharmacokinetic service and consultation order is entered by the physician. The resident pharmacist then fills in the pharmacokinetic service records with the patient history and administration plan and responds to the consultation. Clinical data were obtained from review of these pharmacy records and electronic medical records(EMR) as follows: age; sex; co-morbidities; laboratory and procedural findings;sequential organ failure assessment (SOFA) score; ECMO indication and ECMO type; vancomycin pharmacokinetic parameters including volume of distribution, elimination rate constant k, clearance, vancomycin dosage, duration, and vancomycin concentrations. All patient records/information were anonymized and de-identified prior to analysis in this study. Vancomycin concentrations were quantified using fluorescence polarization immunoassay(COBAS INTEGRA Vancomycin, Roche, Germany). Lower limit of quantification is 1.39 mcg/ml and coefficients of variations (CV %) are 1.6–3.1%. The initial trough level indicates the first level obtained prior to the third dose, and maintenance levels were measured at steady state(usually 5th dose). Every case had at least two vancomycin concentration samples evaluated, one in the early time (within 3rd dose) and another in the steady state (around 5th dose). Trough level was checked right before the next dose administration. Infusion time is 1hr for the dose up to 1g and 2hours over 1g. Reported vancomycin concentration, level check time and infusion start and ending times were all recorded in EMR by nurse and the pharmacokinetic service records by pharmacist.

**Fig 1:**
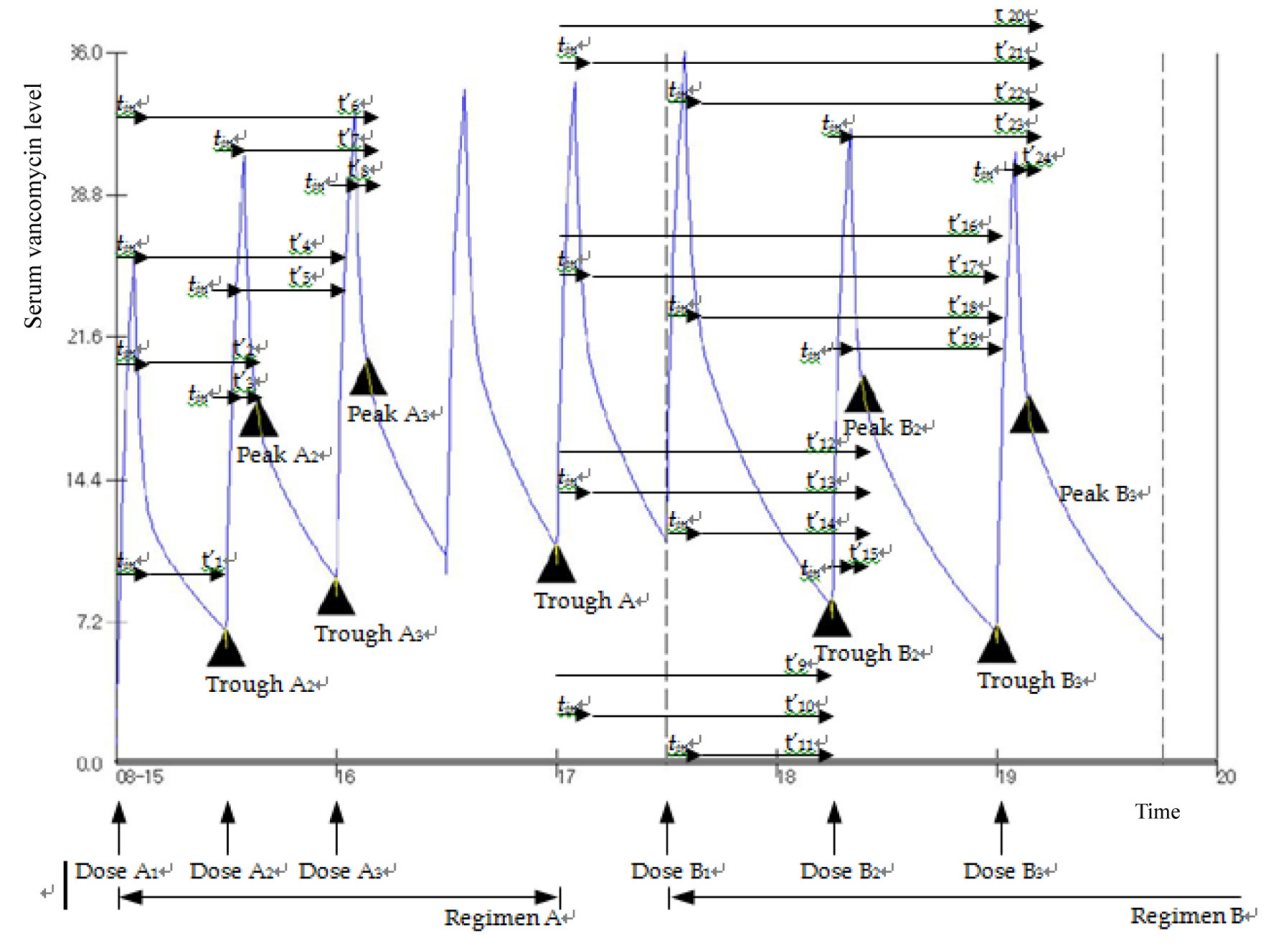
Time and serum vancomycin level changes.

The pharmacokinetic parameters mentioned above and dosage modifications were calculated using short infusion model below[7](Fig 1):

1) Non steady state

$$\mathrm{Trough}\;A_{3}=\frac{\frac{\mathrm{Dose}A}{t_{in}}}{Cl}(1-e^{-kt_{in}})(e^{-kt_{4}^{\prime }}+e^{-kt_{5}^{\prime }})$$

$$\mathrm{Trough}\;B_{2}=(\mathrm{Trough}\;A\times e^{-kt_{9}^{\prime }})+\frac{\frac{\mathrm{Dose}\;A}{t_{in}}}{Cl}(1-e^{-kt_{in}})(e^{-kt_{10}^{\prime }})+\frac{\frac{\mathrm{Dose}\;B}{t_{in}}}{Cl}(1-e^{-kt_{in}})(e^{-kt_{11}^{\prime }})$$

2) Steady state

$$\mathrm{Trough}=\frac{\frac{\mathrm{Dose}/t_{in}}{Cl}}{\left(1-e^{-k\tau }\right)}(1-e^{-kt_{in}})(e^{-k(\tau -t_{in})})$$

Elimination rate constant (K) was calculated by iteration substituting numbers around population parameters. First, we started with population parameter K of similar patient age/ICU group. And find the closest number K to get the exact trough level.

Clearance (Cl, L/hr) = Vd x K

Volume of Distribution (Vd, L) = 0.17 x (age in yr) + 0.22 x (bwt in kg) +15^[^ ^7^ ^]^

Half -life = 0.693/K

Fig 1 is just a mimetic diagram presenting vancomycin serum concentration changes following short infusion administration suggested to help to understand the short infusion model equations above.

We calculated Vd using the equation above because not all the patients had peak concentration value to derive individual Vd because only trough level was routinely monitored. Our study was retrospective so we used only already existing data. Calculation of all of the pharmacokinetic data was performed and evaluated by pharmacists with more than five years of experience, and any ambiguous issues were discussed with daily ICU round members including intensivists, clinical pharmacists, resident physicians, and nurses. After calculating pharmacokinetic parameters, we evaluated if the case reached the target range in the initial period of treatment (within the fourth dose). The pharmacists then recommended a maintenance dosing regimen to the physician through the clinical pharmacokinetic service. The vancomycin target range was set individually by indication according to the 2009 Infectious Diseases Society of America vancomycin dosing guideline[4]. The average time needed to enter the therapeutic range was calculated.

### Statistical analysis

Comparisons of vancomycin dosingfrequency and daily dose between initial period and maintenance period were made using a paired t-test. Comparisons between continuous variables were made using the t-test or the Wilcoxon rank-sum test when applicable. Categorical data were tested using Fisher’s exact test or the Chi-square test.Statistical analyses were performed using SPSS Statistics Version 21 (IBM, Armonk, NY, USA).All tests were two-tailed, and *p*<0.05 was considered statistically significant.

## Results

### Baseline characteristics

During the study period, we ultimately analyzed a total of 80 patients [ECMO group: 20 patients (17 males), control group: 60 patients (38 males)]. Baseline characteristics are shown in Table 1. Compared with patients in the control group, those in ECMO group were overall high-risk subjects despite younger age. Themost common cause of ECMO implantation was respiratory failure. Eleven veno-venous ECMO type and eight veno-arterial ECMO type were included. Because we excluded patients who had acute and chronic renal disease with ongoing renal replacement therapy, creatinine clearances were above 50 ml/min except only one patient in ECMO group. Vancomycin was administered for ventilator-associated pneumonia in 12 patients, for catheter-related infection in five patients, and as an empiric antibiotic for possible MRSA infection while waiting for culture identification of the infective organism in three patients.

**Table 1:** Baseline characteristics.

Variables	ECMO(n = 20)	Control (n = 60)	*P*value
Age, yrs	50.10 ± 16.21	66.90 ± 10.73	<0.001
Sex (male)	17 (85.0)	38 (63.3)	0.070
Diabetes mellitus	3 (15.0)	16 (26.7)	0.373
Hypertension	5 (25.0)	27 (45.0)	0.114
SOFA score	9.95 ± 4.16	5.72 ± 3.78	<0.001
Creatinine, mg/dL	1.02 ± 0.65	0.90 ± 0.45	0.417
Urine output per day, mL	3065 ± 1436	2105 ± 1236	0.012
Cause of ECMO implantation		NA	NA
Cardiogenic shock	5 (25.0)	NA	NA
Respiratory failure	12 (60.0)	NA	NA
Septic shock	3 (15.0)	NA	NA
ECMO type		NA	NA
Veno-venous	11 (55.0)	NA	NA
Veno-arterial	8 (40.0)	NA	NA
Veno-venous to Venoarterial-venous	1 (5.0)	NA	NA

ECMO, extracorporeal membrane oxygenation

SOFA, Sequential Organ Failure AssessmentValues are mean ± standard deviation or n (%).

### Vancomycin pharmacokinetics

**Fig 2:**
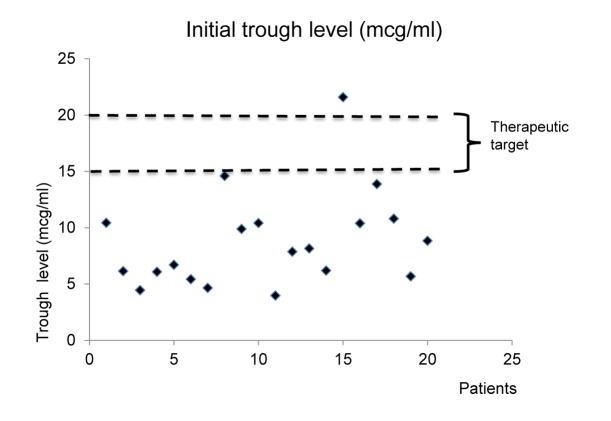
Vancomycin trough levels in initial phase in ECMO group.

**Fig 3:**
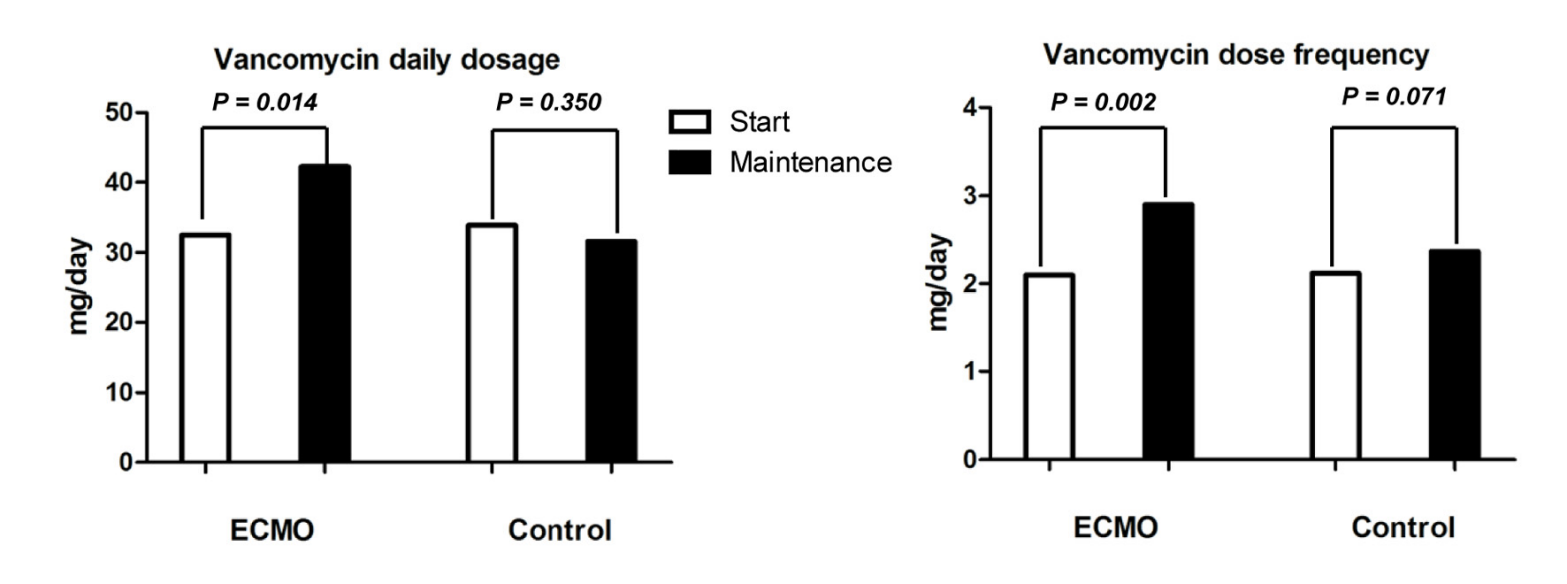
Differences of vancomycin daily total dosage (A) and dosing frequency (B) between initial and maintenance phase.

Among the 20 patients in ECMO group, the vancomycin therapeutic target range was set at 15–20 mcg/ml in 19, and only one patient was suitable for a lower target range of 10–15 mcg/ml. Sixteen patients (80.0%) received an initial intravenous dose of 1.0 g, followed by 1.0 g every 12 hours. The mean starting daily dose was 32.54 mg/kg, and the dosing frequency was 2.10 ± 0.72 times/day. The mean elimination rate constant (K) was 0.12± 0.04 h^-1^, clearance(CL) was 4.62 L/hr, and volume of distribution (Vd) was 0.65 L/kg. The vancomycin clearance/creatinine clearance (Clcr) ratio was 0.90. Pharmacokinetic parameters and clinical parameters of ECMO group are shown in Table 2. The mean initial trough level was as low as 8.80 mcg/ml with a difference of 5.95 mcg/ml compared with the lower limit of the vancomycin target range. Of 20 cases, only one (5%) reached the target range before the fourth dose, and the others were all at sub-therapeutic levels during the initial phase (Fig 2). After the fourth dose, the mean daily dose was increased to 42.24 mg/kg (*p* = 0.014) and the dosing frequency was increased to 2.90 ± 0.97 times/day. Both of these changes from starting dosing patterns to maintenance dosing patterns were statistically significant (*p* = 0.002) (Fig 3). Meanwhile, in control group, 40 patients(66.67%) got subtherapeutic vancomycin level before fourth dose and there was significant difference between ECMO and control groups (*p* = 0.013). Contrast to ECMO group, vancomycin dosingfrequency and total daily dose were not significantly increased after clinical pharmacokinetic services(from 2.11 ± 0.69 to 2.37 ± 0.86 times/day, *p* = 0.071 and from 33.91 ± 11.85 to 31.61 ± 17.50 mg/kg, *p* = 0.350) in the control group. Comparison of vancomycin pharmacokinetics between ECMO and control groups were presented in S1 Table. There were no significant differences in Vd, K and CL between the two groups. The average time to achieve the target trough was significantly longer in ECMO group than control group (84.59 hours vs 57.41 hours, *p* = 0.013). Comparison of pharmacokinetic parameters between veno-venous and veno-arterial ECMO were presented in S2 Table.

**Table 2:** Pharmacokinetic and clinical parameters.

N = 20	ECMOType	Vd (L/kg)	Kinitial	Ksteady state	CLInitial(L/h)	CLsteady state(L/h)	Clcr(L/h)	VancomycinCL/Clcrinitial	Vancomycin CL/Clcsteady state	Leukocyte	CRP	Bodytemperature	Weaningsuccess	Survival todischarge
1	VV	0.689	0.180	0.144	7.344	5.875	7.306	1.005	0.804	23.48	17.78	39.7	No	No
2	VAV	0.690	0.138	NA	5.106	NA	10.860	0.470	NA	18.63	1.84	37.3	No	No
3	VV	0.600	0.173	0.144	6.574	5.472	5.303	1.240	1.032	6.85	1.24	37	Yes	Yes
4	VV	0.695	0.150	0.160	5.505	5.872	10.080	0.546	0.583	21.48	1.26	38	No	No
5	VV	0.586	0.139	0.125	5.699	5.125	4.974	1.146	1.030	17.21	0.15	38.1	Yes	No
6	VV	0.694	0.155	0.135	5.766	5.022	6.600	0.874	0.761	26.36	8.77	38.1	Yes	Yes
7	VV	0.639	0.070	0.180	2.506	6.444	1.484	1.688	4.341	17.87	2.04	37.2	Yes	No
8	VV	0.560	0.070	0.070	2.528	2.528	6.458	0.391	0.391	30.33	15.92	35.7	Yes	Yes
9	VA	0.611	0.155	0.155	4.828	4.828	5.980	0.807	0.807	5.1	28.59	37.5	No	No
10	VA	0.549	0.120	0.067	3.648	2.037	7.200	0.507	0.283	8.91	5.87	39.3	Yes	Yes
11	VA	1.000	0.143	0.119	8.437	7.021	5.624	1.500	1.248	22.74	4.77	37.4	Yes	Yes
12	VV	0.553	0.127	0.122	4.623	4.441	5.836	0.792	0.761	29	7.99	38.6	Yes	No
13	VV	0.624	0.125	0.094	5.125	3.854	4.456	1.150	0.865	18.75	21.08	38.4	Yes	No
14	VV	0.602	0.110	0.110	5.500	5.500	8.897	0.618	0.618	18.49	6.72	36.8	No	No
15	VA	0.799	0.065	0.067	2.165	2.231	3.951	0.548	0.565	17.55	3.86	38.4	Yes	Yes
16	VA	0.530	0.116	0.070	3.480	2.100	3.095	1.124	0.678	16.23	11.72	38.3	Yes	Yes
17	VA	0.721	0.037	0.030	1.443	1.170	3.576	0.404	0.327	26.96	7.5	37.9	No	No
18	VA	0.505	0.097	0.093	4.152	3.980	4.624	0.898	0.861	20.07	17.91	38	Yes	Yes
19	VV	0.583	0.070	0.070	2.940	2.940	3.206	0.917	0.917	5.38	29.93	37.3	No	No
20	VA	0.661	0.120	0.072	5.040	3.024	3.927	1.283	0.770	14.56	12.15	37.8	Yes	Yes
Mean/success %		0.645	0.118	0.101	4.620	4.182	5.672	0.895	0.929	-	-	-	65%	45%

Vd, volume of distribution

K, elimination rate constant

CL, vancomycin clearance

Clcr, creatinine clearance

Vancomycin CL/Clcr, vancomycin clearance/creatinine clearance

CRP, C-reactive protein

NA, not available

VV, Veno-venous

VA, Veno-arterial

VAV, Venoarterial-venous

## Discussion

In the present study, we evaluated the appropriateness of the current dosing strategy for vancomycinin our institution based on total body weight and creatinine clearance in adult patients on ECMO. Most of study patients received 1g q12hours regimen as an initial dosing because 15–20mg/kg q12hours ~ q8hours regimen is recommended based on guidelines and our usual ICU patients’ body weights are spread over 50~60kg. Our data showed that the current dosing strategy of our institution is not sufficient to achieve the target trough in the initial period, despite the elimination rate constant for vancomycin was within similar range with population parameter of non ECMO patients.

Because a large proportion of ICU patients require a therapeutic level higher than 15 mcg/ml, it is concern that the initial vancomycin level remains lower than the target range and that a long time is required to reach a steady state expected to be therapeutic[4, 8]. In particular, the hydrophilicity of vancomycin leads to variation in the volume of distribution, and this factor is related to the initial vancomycin serum concentration. It can be inferred that devices that replace large amounts of plasma volume such as ECMO or continuous renal replacement therapy (CRRT) may cause hemodilution, and that the initial plasma concentration of vancomycin might be relatively low during the initial phase of use with these devices[2, 9]. Previous studies have reported that antibiotic dosing schedules were frequently insufficient due to hemodilution such as CRRT, especially for hydrophilic antibiotics like vancomycin or meropenem, and insisted that a loading dose and higher maintenance dosage are needed[10, 11].Moreover, because up to 90% of vancomycin is excreted through the kidney, the maintenance dosage and serum level are both related to renal function. Accordingly, we excluded patients who were treated with renal replacement therapy in order to avoid the influence of differences in excretion and to allow us to investigate only the effects of ECMO on vancomycin initial serum level.Of 20 patients in this study, 19 patients (95%) had a sub-therapeutic level of vancomycin in the initial phase, and the mean initial trough level was less than 10 mcg/ml. In general, the initial drug plasma concentration will inevitably be low because it starts from a serum level of zero; the period of low concentration should be reduced in order to assure positive outcome. This result was much higher failure percentage compared to previously published data of non-ECMO patients. Kim *et al*. reported that initial steady state vancomycin level was sub-optimal in more than 50% of pediatric patients [12] and, in particular, when the target range was higher than 15 mcg/ml, only 20.3% of the study population was within the range. In another study performed in 2012 [5], Parekh *et al*. reported vancomycin dosing and initial trough levels in critically ill patients. Their data involving an average initial dose of 14.2 mg/kg showed that 44% of patients had an initial trough level less than 10 mcg/ml. Other published data with dosing regimen of 1g every 12 hours like our study also said that about 58~70% of patients without ECMO failed to reach the recommended therapeutic serum trough concentration [13, 14].Moreover, the average time to achieve a therapeutic trough was 83 hours. Similarly, our study required 84.59 hours to reach the target range and that indicates significantly longer time than control group. That means that the plasma vancomycin level remained below the sub-therapeutic level for more than three days. Kumar *et al*.[6]reported the importance of effective antimicrobial administration in the early phase of septic shock and inferred that earlier achievement of the target level is needed.

Among the vancomycin pharmacokinetic parameters, the elimination constant and clearance in ECMO patients in our study were similar to those in the general population (non ECMO patients).However, because the volume of distribution was fixed by a formula because of the lack of records on peak level, we could not account for the administration of ECMO, which might induce changes in the volume of distribution in individual patients. Both the daily starting dosage and dosing frequency showed a statistically significant increase between initial and maintenance dosingin ECMO group. Accordingly, our results suggest that an initial larger dosage through loading dose administration or shortened initial dosing interval will allow the vancomycin level to reach the target range earlier[4, 15].

Our study has several limitations. The nonrandomized nature of the registry data could have resulted in selection bias. Because of the retrospective character of this study, we could not use the vancomycin peak level to calculate the exact volume of distribution because peak level is not commonly used when performing clinical pharmacokinetic services and there were lots of monitoring skips. If we could calculate the volume of distribution on an individual level reflecting change due to ECMO, it would be easier to review whether the loading dose is helpful. Finally, sixteen patients (80.0%) received an initial dose of 1.0 g, followed by 1.0 g every 12hours so it is difficult to compare impacts of the dosing strategy on clinical outcomes and pharmacokinetics. Therefore, large-scale prospective trials are needed to clarify the appropriateness of initial vancomycin dosing in critically ill patients undergoing primary ECMO.

## Conclusions

The current dosing strategy utilized at our ICU (15 to 20 mg/kg/dose every 8 to 12 hours) was not sufficient to achieve the target trough in the initial period in most patients receiving ECMO, despite the elimination rate constant for vancomycin was within similar range with population parameter of non ECMO patients. Special attention to the therapeutic trough concentration during the initial period is warranted in critically ill patients on ECMO.

## Supporting Information

S1 Table Comparison of vancomycin pharmacokinetics between ECMO and control groups.Vd, volume of distribution; K, elimination rate constant; CL, vancomycin clearance; Clcr, creatinine clearance; Vancomycin CL/Clcr, vancomycin clearance/creatinine clearance (DOCX)

S2 Table Comparison of vancomycin pharmacokinetics between VV ECMO and VA ECMO. Vd, volume of distribution; K, elimination rate constant; CL, vancomycin clearance; Clcr, creatinine clearance; Vancomycin CL/Clcr, vancomycin clearance/creatinine clearance
(DOCX)
