# Supplementary material for: Trough Concentrations of Vancomycin in Patients Undergoing Extracorporeal Membrane Oxygenation
Source: PLoS One. 2015 Nov 6;10(11):e0141016. doi: 10.1371/journal.pone.0141016 (PMC4636270; doi:10.1371/journal.pone.0141016)
Supplement: S1 Table — (DOCX) [file pone.0141016.s001.docx]

**S1 Table.** Comparison of vancomycin pharmacokinetics between ECMO and control groups

| Variables | ECMO  (n=20) | Control  (n=60) | *P* value |
| --- | --- | --- | --- |
| Vd (L/kg) | 0.65 ± 0.11 | 0.68 ± 0.11 | 0.292 |
| K_initial | 0.12 ± 0.04 | 0.11 ± 0.07 | 0.624 |
| K_steady state | 0.10 ± 0.05 | 0.08 ± 0.04 | 0.076 |
| CL_Initial(L/h) | 4.62 ± 1.79 | 4.31 ± 3.17 | 0.682 |
| CL_steady state (L/h) | 3.97 ± 1.90 | 3.13 ± 1.54 | 0.05 |
| Clcr (L/h) | 5.67 ± 2.38 | 3.26 ± 1.23 | <0.001 |
| Vancomycin CL/CLcr_initial | 0.90 ± 0.37 | 1.40 ± 0.84 | 0.011 |
| Vancomycin CL/CLcr_steady state | 0.93 ± 0.86 | 1.02 ± 0.49 | 0.552 |

Vd, volume of distribution; K, elimination rate constant; CL, vancomycin clearance; Clcr, creatinine clearance; Vancomycin CL/Clcr, vancomycin clearance/creatinine clearance
