# Supplementary material for: Trough Concentrations of Vancomycin in Patients Undergoing Extracorporeal Membrane Oxygenation
Source: PLoS One. 2015 Nov 6;10(11):e0141016. doi: 10.1371/journal.pone.0141016 (PMC4636270; doi:10.1371/journal.pone.0141016)
Supplement: S2 Table — Vd, volume of distribution; K, elimination rate constant; CL, vancomycin clearance; Clcr, creatinine clearance; Vancomycin CL/Clcr, vancomycin clearance/creatinine clearance (DOCX) [file pone.0141016.s002.docx]

**S2 Table.** Comparison of vancomycin pharmacokinetics between VV ECMO and VA ECMO

| Variables | VV ECMO (n=12) | VA ECMO  (n=8) | *P* value |
| --- | --- | --- | --- |
| Vd (L/kg) | 0.63 ± 0.54 | 0.67 ± 0.17 | 0.384 |
| K_initial | 0.13 ± 0.39 | 0.11 ± 0.04 | 0.300 |
| K_steady state | 0.11 ± 0.05 | 0.08 ± 0.04 | 0.179 |
| CL_Initial(L/h) | 4.94 ± 1.55 | 4.15 ± 2.13 | 0.350 |
| CL_steady state (L/h) | 4.42 ± 1.84 | 3.30 ± 1.91 | 0.203 |
| Clcr (L/h) | 6.29 ± 2.73 | 4.75 ± 1.40 | 0.161 |
| Vancomycin CL/CLcr_initial | 0.90 ± 0.37 | 0.88 ± 0.40 | 0.913 |
| Vancomycin CL/CLcr_steady state | 1.10 ± 1.09 | 0.69 ± 0.31 | 0.322 |

Vd, volume of distribution; K, elimination rate constant; CL, vancomycin clearance; Clcr, creatinine clearance; Vancomycin CL/Clcr, vancomycin clearance/creatinine clearance
